# Endoscopic treatment of early leaks and strictures after laparoscopic one anastomosis gastric bypass

**DOI:** 10.1186/s12893-020-0686-2

**Published:** 2020-02-21

**Authors:** Fadi Younis, Mati Shnell, Nathan Gluck, Subhi Abu-Abeid, Shai Eldar, Sigal Fishman

**Affiliations:** 1 Obesity Service, Department of Gastroenterology and Liver Disease, Tel Aviv Sourasky Medical Center, affiliated with Sackler School of Medicine, Tel Aviv University, 6 Weizmann St, Tel Aviv, Israel; 2 Bariatric Unit, Department of Surgery, Tel Aviv Sourasky Medical Center, Tel Aviv, Israel

**Keywords:** Laparoscopic one anastomosis gastric bypass, Postoperative complications, Bariatric endoscopy, Stents, Dilation

## Abstract

**Background:**

Laparoscopic one anastomosis gastric bypass has become a prominent bariatric procedure. Yet, early and late complications, primarily leaks and strictures, are not uncommon. This study summarizes our experience with endoscopic treatment of laparoscopic one anastomosis gastric bypass complications.

**Methods:**

This is a retrospective study of consecutive patients referred to our hospital from 2015 to 2017 with post laparoscopic one anastomosis gastric bypass complications. Therapy was tailored to each case, including fully covered self-expandable metal stents, fibrin glue, septotomy, internal drainage with pigtail stents, through-the-scope and pneumatic dilation. Success was defined as resuming oral nutrition without enteral or parenteral support or further surgical intervention.

**Results:**

Nine patients presented with acute or early leaks: 5 (56%) had staple-line leaks, 3 (33%) had anastomotic leaks and 1 (11%) had both. All were treated with stents. Adjunctive endoscopic drainage was applied in 4 patients (44%). Overall 5 patients (56%) with acute/ early leaks recovered completely, including all 3 patients with anastomotic leak and the patient with both leaks but only 1/5 with staple line leak (20%). Complication rate in the leak group reached 22%. Eight patients presented with strictures, 7 at the anastomosis and one due to remnant stomach misalignment. All anastomotic strictures were dilated successfully. However, the patient with the pouch stricture required conversion to Roux-en-Y gastric bypass after 3 failed attempts of dilation.

**Conclusion:**

Endoscopic treatments of laparoscopic one anastomosis gastric bypass complications are relatively effective and safe. Anastomosis-related complications are more amenable to endoscopic treatment compared to staple line leaks.

## Background

Morbid obesity has become a major global health threat that leads to severe morbidity including diabetes, hypertension, obstructive sleep apnea, degenerative joint disease and cardiovascular diseases. To date, bariatric surgery is the most effective intervention for weight reduction and remission of associated comorbidities [1]. Laparoscopic one anastomosis gastric bypass (LOAGB), introduced in 1997 [2], is gaining popularity and has become the fourth most performed surgery in Europe and the Asia/Pacific area [1, 3]. Results with LOAGB in terms of weight loss and resolution of comorbidities have been promising [4–6]. Still, early and late complications occur at an estimated rate of 3.1 and 10% respectively [7]. Potential adverse events include postoperative or chronic leaks and strictures. Endoscopic management of postoperative leaks is challenging and constantly evolving with no clear guidelines. The aim of endoscopic treatment is to divert gut secretion away from the leak site to allow fistula healing and resuming oral nutrition as early as possible. These goals may be achieved by deploying fully covered self-expanding metal stents (FC-SEMS) [8–13]. Other strategies including over the scope clips, fibrin glue, endoscopic suturing and intragastric drainage (IGD) with double pigtail stent have been all described with variable success rate [9, 14–17]. In cases of chronic leak with perigastric collection formation, the preferred and effective endoscopic approach is to enable adequate drainage of the perigastric collection into the stomach by dissecting the septum separating the two cavities (septotomy) [18] or by inserting double pigtail stent. Post-surgical strictures are treated with endoscopic dilation. While anastomotic strictures are amenable to through the scope (TTS) dilation with high success rates [19, 20], post laparoscopic sleeve gastrectomy (LSG) strictures due sleeve misalignment are more difficult to treat. In these cases pneumatic dilation is preferred [21]. The aim of this study was to summarize our experience in treating post LOAGB complications amenable to endoscopic treatment, namely leaks and strictures.

## Methods

This was a retrospective study of consecutive patients referred to our department with post LOAGB leaks or strictures between August 2015 and October 2017**.** The diagnosis was based upon symptoms, imaging and endoscopic studies. Patients were managed by a multidisciplinary team that included bariatric surgeons, gastroenterologists from the bariatric endoscopy service, invasive radiologists and nutritionists.

### Postoperative leaks

**Fig. 1:**
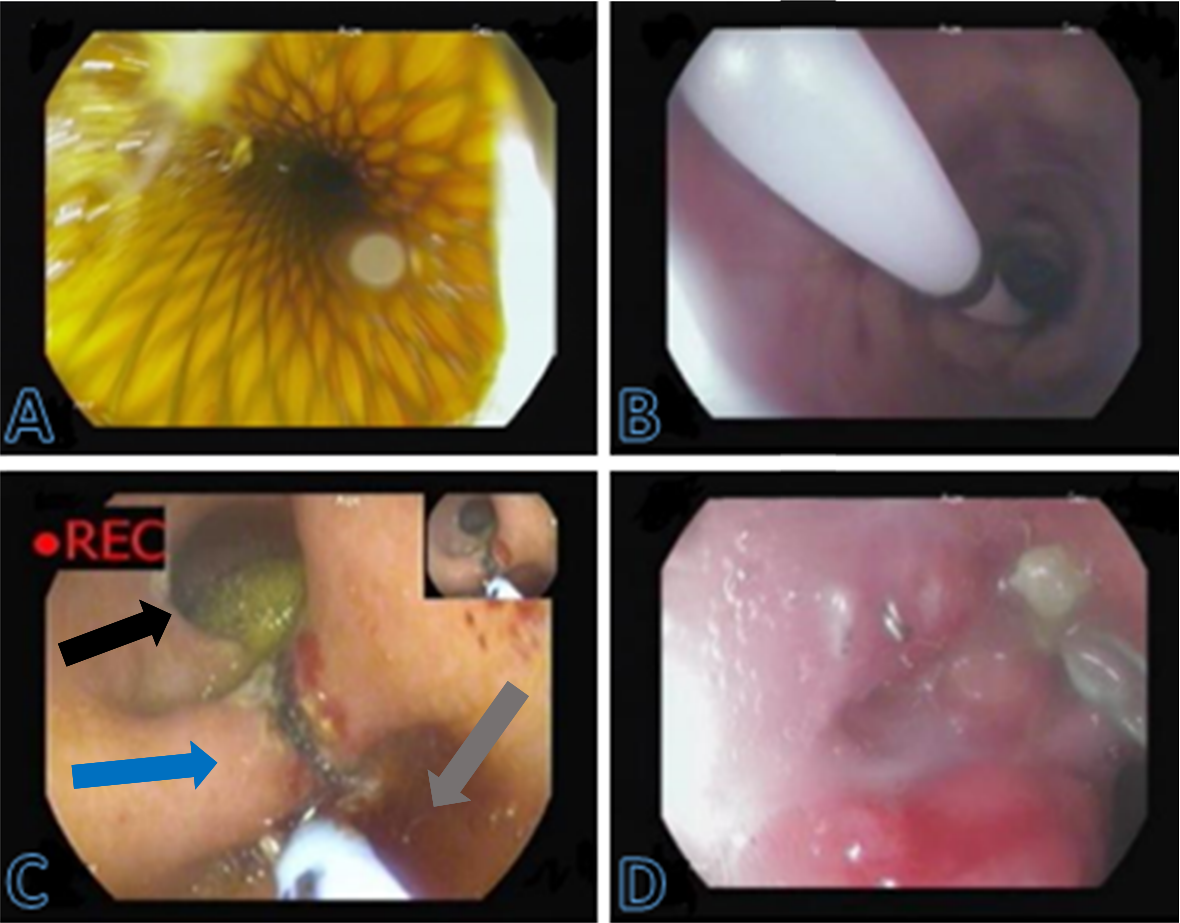
Treatment of a staple-line leak. **a**. Treatment at the acute phase with stent deployment. **b**. Treatment at the late phase with pneumatic dilation of the remnant stomach. **c**. Septotomy performed to unify the perigastric collection and the remnant stomach cavity, views showing the perigastric space (black arrow), remnant stomach lumen (gray arrow) and septum (blue arrow). **d**. A healed fistula

In all cases fully covered stents (Esophageal Mega stent, length 23 mm, diameter 24 mm, Teawoong, Seoul, Korea) were introduced over a stiff guidewire (38-in. Amplatz Super Stiff, Boston Scientific, Marlborough, Mass, USA) with fluoroscopic guidance, under conscious sedation. The proximal third of the stent was located above the gastroesophageal junction and the distal end was placed in the efferent limb. Fluoroscopy was performed 1 day after the procedure to confirm correct positioning of the stent and resolution of the leak after which patients gradually resumed a soft oral diet. Our target duration of treatment was 2–6 weeks and was individualized by drainage output when a drain was placed, the nature of the fluid and patient tolerability. In cases where the leak evolved to a late or chronic form (> 6 or 12 weeks, respectively), internal drainage was achieved by septotomy (see Introduction) or pigtail insertion into the collection cavity. Septotomy was performed by incising the septum with argon plasma coagulation (Erbe Vio 200 D; forced coagulation, 70 W, 2 L/min) along the staple-line towards the base of the perigastric cavity, which was endoscopically identified. Importantly, the incision of the septum never exceeded the base of the cavity. As a precaution, the incision was performed gradually, with a maximal incision depth of 5–10 mm during each session until the cavity was entered. Patients resumed an oral liquid diet once adequate intragastric drainage had been achieved. Double pigtail stent with a diameter of 7 Fr and length of 7 cm (Cook Medical) were inserted under fluoroscopy into persistent residual sinuses. All procedures were performed using CO2 insufflation (Fig. 1).

### Postoperative strictures

All patients were treated with balloon dilation. Anastomotic strictures were treated with TTS balloon dilation (CRE™ Balloon Dilation Catheters; Boston Scientific, Natick, MA, USA). The balloon was gradually inflated up to maximal diameter of 20 mm. The procedure was repeated as needed. Remnant stomach misalignment resulting in mid gastric kink was treated with pneumatic balloon dilation (Rigiflex II balloon; Boston Scientific, Natick, MA, USA). The procedure was performed under direct vision and fluoroscopy with continuous inflation up to a maximal diameter of 30 mm and pressure of 20 PSI in each session. The balloon was held maximally inflated within the stricture for 1 min. When two consecutive procedures did not alleviate the symptoms the patient was referred to surgery.

Success was defined as resuming oral nutrition without enteral or parenteral support or further surgical intervention.

### Statistical analysis

Continuous variables were tested for normality. Normally distributed parameters were described as mean ± standard deviation, where skewed parameters were describes as median (interquartile range, IQR), and categorical as N (%) using SPSS (IBM SPSS Statistics for Windows, Version 21.0. 2012, Armonk, NY: IBM Corp.)

## Results

Seventeen patients were referred to our clinic, 9 with postoperative leaks and 8 with postoperative strictures.

### Postoperative leak group

Nine patients were included in this group, all were presented less than 4 weeks after surgery, 5/9 male, with a mean age of 41 ± 11 years (range 23–57) and pre-operative mean body mass index (BMI) of 44 ± 8 (kg/m2). Interestingly, six patients (67%) had revisions from previous bariatric surgery including laparoscopic adjustable gastric banding (LAGB), silastic ring vertical gastroplasty (SRVG) and laparoscopic sleeve gastrectomy, 3 of which had two previous operations (Table 1). Neither of the patients developed frank peritonitis or needed another surgical procedure. All cases were diagnosed by computed tomography. Endoscopic management applied in each patient and outcomes are depicted in Table 2. Eight patients (89%) had an early leak (1–6 week) and one had an (11%) acute leak (< 1 week). Five patients (56%) presented with a proximal staple-line leak (at the angle of His), 3 (33%) with an anastomotic leak and 1 (11%) with leaks at both sites. Median time between surgery and first endoscopy was 12 days (IQR 10–19) and the median number of therapeutic endoscopic sessions was 3 (IQR 2–6). All patients were treated with fully covered stents for a median period of 26 days (IQR 11–30). Additional endoscopic treatment was required in 4 patients (45%). Three of them needed ancillary dilation. The first needed a single TTS balloon dilation (15 mm) of an anastomotic stricture. The second needed a single pneumatic balloon (30 mm) dilation of a mid-pouch stricture. The third developed a late stricture at the esophageal gastric junction, treated with gradual TTS dilation up to 20 mm. Two patients with staple line leak evolved to late fistula and needed additional drainage procedures including septotomy, double pigtail stent insertion and tissue glue (Table 2).

**Table 1:** Patient demographics and surgical details of postoperative leak group

Patient number	BMI (Kg/m2)	Previous operation
1	37	None
2	NA	LAGB + LSG
3	38	LAGB + LSG
4	46	SRVG
5	38	None
6	40	None
7	60	LAGB × 2
8	NA	LAGB
9	NA	LSG

*BMI* Body mass index, *LAGB* Laparoscopic adjustable gastric banding, *LSG* Laparoscopic sleeve gastrectomy, *NA* Not available, *SRVG* Silastic ring vertical gastroplasty

**Table 2:** Endoscopic leak management and outcomes

Patient number	Leak site	Days from surgery to leak diagnosis (type of leak)	Days from surgery to first endoscopy	Number of therapeutic endoscopies	Stent duration (days)	Number of stent replacements/ repositions	Additional treatment	Result
1	Anastomosis Staple line^a^	9 (early)	10	6	36	–	GEJ. Dilation	Success
2	Staple line^a^	14 (early)	21	7	30	1	Anastomosis Dilation Septotomy	Success
3	Staple line^a^	7 (early)	10	12	25	4	Septotomy Pigtail Tissue glue	Failure
4	Anastomosis	9 (early)	10	3	8	–	No	Success
5	Anastomosis	26 (early)	6	2	19	–	No	Success
6	Staple line^a^	14 (early)	17	2	1	–	No	Failure
7	Staple line^a^	5 (acute)	29	3	30	1	Mid Pouch. Dilation	Failure
8	Anastomosis	10 (early)	12	2	26	–	No	Success
9	Staple line^a^	11 (early)	16	1	14	–	No	Failure

*GEJ* Gastroesophageal junction

^a^At the angle of His

Overall, 5 of 9 patients (56%) were successfully treated as defined by weaning from total parenteral nutrition, resuming oral diet, removal of intra-abdominal draining tubes, and avoidance of further surgical intervention with a follow up of approximately 6 months (Table 2). All 3 patients with anastomotic leak had a favorable outcome. However only 1 of 5 patients with staple line leak had a favorable outcome. Of note, the patient with both anastomotic- and staple line-leaks recovered.

Two patients were referred to Roux-en-Y- gastric bypass (RYGB) conversion. One patient needed urgent laparotomy after 2 weeks of treatment due to stent migration and ileum perforation. Interestingly, this patient did not require further treatment for his staple-line leak. The forth patient died due to respiratory failure secondary to severe pneumonia probably not related to the endoscopic procedure. Of note, 3 of 4 failed patients (75%) had previous bariatric surgery.

### Postoperative stricture group

Eight patients were diagnosed with stricture based upon symptoms of vomiting and excessive weight loss**.** All of them were female with a mean age of 49 ± 14 years (range 24–65) and preoperative mean BMI of 39 ± 9 (kg/m2). Four patients (50%) had previous bariatric surgery including LAGB and LSG (Table 3). Stricture site and mode of treatment are depicted in Table 4. Seven patients (88%) presented with anastomotic stricture and one patient (12.5%) with a mid-pouch kink. The median time between surgery and the first endoscopic dilation was 63 days (IQR 37–140)**,** and the median number of therapeutic endoscopic dilation was 3 (2–4). Anastomotic strictures were TTS-dilated with a maximal balloon diameter of 20 mm and the pouch stricture was pneumatically dilated with a balloon diameter of 30 mm. Overall success rate was 88%. All 7 patients with anastomotic stricture reported significant clinical improvement with a follow up of 6 months after the last procedure (Table 4). The patient with the pouch kink was defined as a treatment failure due to persistent vomiting and weight loss after 3 attempts of pneumatic dilation and finally underwent conversion to formal RYGB surgery.

**Table 3:** Patient demographics and surgical details of postoperative stricture group

Patient number	BMI (kg/m2)	Previous operation
1	37	No
2	46	No
3	NA	LSG
4	39	No
5	NA	LAGB
6	48	LSG
7	23.3	LSG
8	40	No

*BMI* Body mass index, *CT* Computed tomography, *LAGB* Laparoscopic adjustable gastric banding, *LSG* Laparoscopic sleeve gastrectomy, *NA* Not available

**Table 4:** Endoscopic dilation of strictures and outcomes

Patient number	Stricture site	Days from surgery to first dilation	Number of therapeutic dilations	Type of dilation	Maximal size of dilation	Result
1	Anastomosis	147	2	TTS	20 mm	Success
2	Anastomosis	50	5	TTS	18 mm	Success
3	Anastomosis	30	1	TTS	20 mm	Success
4	Anastomosis	33	3	TTS	15 mm	Success
5	Anastomosis	160	4	TTS	15 mm	Success
6	Anastomosis	117	3	TTS	20 mm	Success
7	Pouch	60	3	P	30 mm	Failure
8	Anastomosis	66	3	TTS	18 mm	Success

*P* Pneumatic, *TTS* Through-the-scope

## Discussion

Herein we demonstrate that endoscopic treatment of post-LOAGB leaks and strictures is can be effective and is safe. To our knowledge this is the largest series described to date. In 10 of 17 patients a previous bariatric procedure had been performed (59%), which is a known risk factor for post-surgery complications [22, 23]. Interestingly, LOAGB complications may be divided into anastomosis-related stricture and leaks which resemble those of RYGB [24–26], and staple line leaks and remnant stomach axis deviation (kink) which are similar to those of LSG [27–29]. Our data show a 100% (7/7) success rate in TTS dilation of anastomotic strictures without any complications at a mean follow up of 6 months, similar to the 93–100% success rate for post RYGB anastomotic stricture dilation [30]. Anastomotic leaks controlled with stent insertion also exhibited a favorable outcome with 100% success (3/3). In contrast, treatment of staple line leaks exhibited a less favorable outcome with a success rate of only 20% (1/5), compared to 65–95% in acute and early leaks after LSG [30]. Of note, two patients evolved to a late type of leak and received ancillary drainage with septotomy and double pigtail stent. Although we achieved excellent control of late and chronic leaks after LSG with this approach [18], currently it succeeded in only one patient. Another patient had remnant stomach misalignment (kink) with failure to dilate it pneumatically. Interestingly there was high rate of treatment failure of staple line-related complications in patients with previous bariatric procedures (60%) which supports the possibility of causality.

A recent study described 46 leaks in one anastomosis gastric bypass out of 2780 operated patients. This series highlights the effectiveness of the endoscopic treatment, as 20% of the primary management was done successfully by this mean [31].

The safety profile of endoscopic treatment could be further improved. Overall, we had two major complications. One case of stent migration resulted in small bowel perforation which necessitated urgent laparotomy. One death occured, however it was not related to the endoscopic procedure.

Our study has several limitations. These include a relatively small group of patients, performance at a single tertiary referral centre, and no control group in the study design. Multicentric trials, with greater sample sizes are necessary.

## Conclusion

Endoscopic treatment of LOAGB complications can be effective and relatively safe. Anastomosis-related complications are more amenable to endoscopic treatment compared to staple line related complications.

## Data Availability

From the corresponding author on reasonable request.

## Abbreviations

BMI: Body mass index
FC-SEMS: Fully covered self-expanding metal stents
IGD: Intragastric drainage
LOAGB: Laparoscopic one anastomosis gastric bypass
LSG: Laparoscopic sleeve gastrectomy
OTSC: Over the scope clips
RYGB: Roux-en-Y- gastric bypass
TTS: Through the scope
